# Microscopic Origin of Strain Hardening in Methane Hydrate

**DOI:** 10.1038/srep23548

**Published:** 2016-03-24

**Authors:** Jihui Jia, Yunfeng Liang, Takeshi Tsuji, Sumihiko Murata, Toshifumi Matsuoka

**Affiliations:** 1 Environment and Resource System Engineering, Kyoto University, Kyoto 615-8540, Japan; 2 International Institute for Carbon-Neutral Energy Research (I2CNER), Kyushu University, Fukuoka 819-0395, Japan

## Abstract

It has been reported for a long time that methane hydrate presents strain hardening, whereas the strength of normal ice weakens with increasing strain after an ultimate strength. However, the microscopic origin of these differences is not known. Here, we investigated the mechanical characteristics of methane hydrate and normal ice by compressive deformation test using molecular dynamics simulations. It is shown that methane hydrate exhibits strain hardening only if the hydrate is confined to a certain finite cross-sectional area that is normal to the compression direction. For normal ice, it does not present strain hardening under the same conditions. We show that hydrate guest methane molecules exhibit no long-distance diffusion when confined to a finite-size area. They appear to serve as non-deformable units that prevent hydrate structure failure, and thus are responsible for the strain-hardening phenomenon.

Methane hydrate is an ice-like crystalline solid in which methane molecules are trapped as guest molecules to stabilize the otherwise unstable hydrogen-bonded network with distinct polyhedral water cages (Fig. 1, see also Fig. S1)^1^,^2^,^3^,^4^. Methane hydrate is abundant in the earth’s permafrost and marine sediments at continental margins. In addition, methane and carbon dioxide hydrates are likely to occur in outer solar systems^5^ and in Polar Regions of Mars^6^, respectively. Because of their enormous worldwide reserves, methane hydrate has attracted significant attention and is considered to be the largest untapped global energy resource^7^,^8^,^9^,^10^. The presence of methane hydrate affects the strength, stability, porosity and flow characteristics of hydrate-bearing sediments. Their destabilization by drilling, production, or environmental temperature and pressure changes may impact sea-floor stability. Furthermore, methane release may pose a serious environmental threat^3^. Huge energy resources, the significance of planets and satellites in the solar system, and the potential environmental threat of methane hydrate have led to experimental laboratory studies on the physical properties of methane hydrate, which can be divided into two groups: (1) pure methane hydrate or other ice compounds^11^,^12^,^13^,^14^,^15^,^16^,^17^,^18^,^19^,^20^; and (2) the influence of mineral grains^20^,^21^,^22^,^23^,^24^. Among them, it is essential to understand the mechanical strength of methane hydrate to predict the stability of its formation during gas decomposition^11^,^12^,^13^,^14^,^15^,^22^.

Strain hardening is a phenomenon in which a compound becomes harder and stronger as it is deformed plastically^25^. From constant-strain-rate compressional deformation experiments in a tri-axial gas deformation apparatus, it was found that methane hydrate exhibits strain hardening to an unusually high degree (>18% strain) compared with normal ice, which displays typical ultimate strength within the first 5–10% of strain^11^,^12^. This strain range even exceeds that of most metals and ceramics^25^. The stoichiometry change (e.g., ex-solution or disproportionation) during plastic deformation may be responsible, or partially responsible, for this strain-hardening behaviour^11^,^12^. However, no detailed microscopic model exists in this regard.

Molecular dynamics (MD) simulation, as a microscopic method, is a powerful way to understand hydrate at the molecular level with linkages to macroscopic phenomena^26^,^27^,^28^,^29^,^30^,^31^,^32^,^33^,^34^. It can be used to deliver insights into problems not well understood by experiments. Few MD studies on hydrate strength behaviour have been reported^20^. This study aimed to investigate the mechanical characteristics, especially strain-hardening behaviour^11^,^12^, of methane hydrate (sI) in contrast with normal ice (Phase Ih) (Fig. 1, see also Fig. S1), based on a response to constant deformation rate tests performed in MD simulations.

## Results

### Elastic and Plastic Regimes: Observation of Strain Hardening

The elastic moduli of methane hydrate and normal ice were evaluated at 250 K and 40 MPa. Methane hydrate has three elastic constants (*C*_11_, *C*_12_, *C*_44_) because it is a cubic system, and normal ice has five independent elastic constants (*C*_11_, *C*_12_, *C*_13_, *C*_33_, *C*_44_) because it is a hexagonal system. Tables S1 and S2 in the supplementary materials list the computed elastic constants that are obtained from the slopes of the stress–strain curves and Table S3 lists the calculated moduli, including the bulk modulus K, the shear modulus G, Poisson’s ratio *v* and Young’s modulus for each material. They were compared with experimental results from Brillouin spectroscopy^18^,^19^ and the ultrasonic pulse transmission method^16^, respectively. It shows that the calculated *C*_11_, *C*_12_, *C*_13_, *C*_33_ bulk moduli and Poisson’s ratio (of normal ice) are slightly overestimated, whereas the calculated *C*_44_, shear moduli and Young’s moduli (of normal ice) are somewhat smaller. For methane hydrate, similar results were obtained. The simulation and experimental results indicate that methane hydrate is slightly more compressible (according to the bulk modulus, *C*_11_, and *C*_33_) but more rigid (according to the shear and Young modulus) than normal ice, which also implies consistency of the data sets. The agreement verifies the applicability of relevant parameters to the simulation results on mechanical properties.

**Figure 1:**
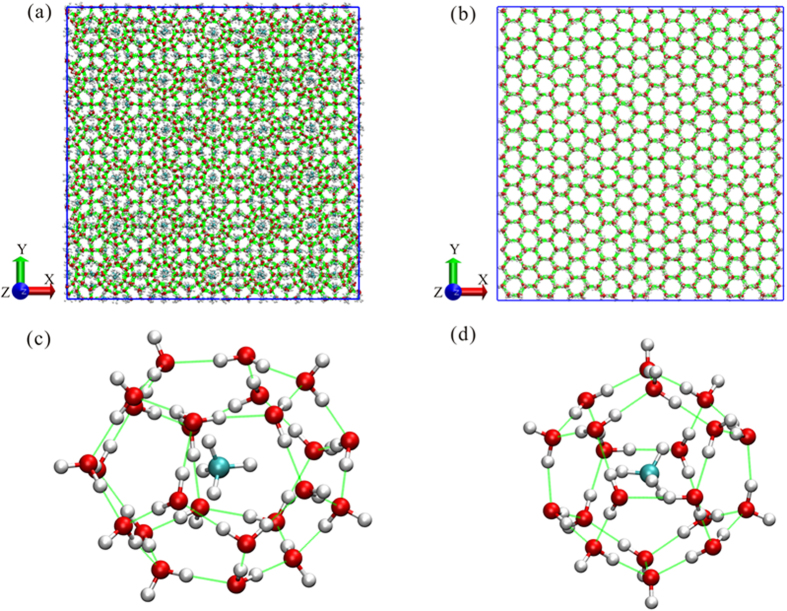
Molecular structures of (**a**) methane hydrate (sI) and (**b**) normal ice (Ih). (**c**) Large (5^12^6^2^) and (**d**) small (5^12^) methane hydrate cages.

**Figure 2:**
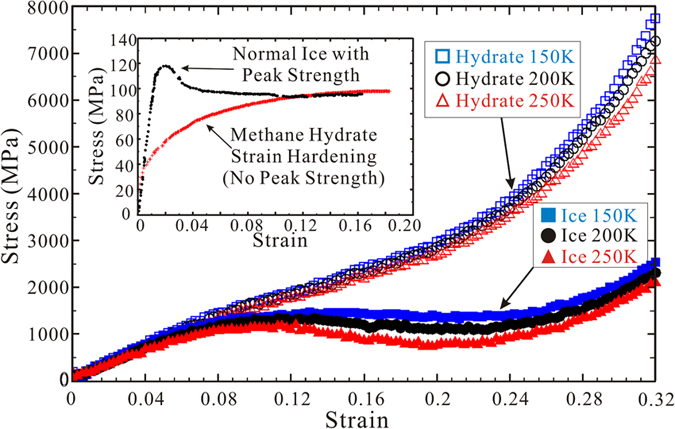
Comparison of stress-strain relationships between methane hydrate and normal ice in the X direction. All simulations were conducted with a strain rate of 5 × 10^9 ^s^−1^ at 40 MPa. The inset of panel (a) is from experimental results^10^,^11^. Experiments were conducted at 160 K and 50 MPa for normal ice and 168 K and 100 MPa for methane hydrate. The strain rate was 3.5 × 10^−5 ^s^−1^.

For methane hydrate and normal ice, the X, Y and Z directions are represented by (1 0 0), (0 1 0) and (0 0 1) and (1 −2 0), (0 1 0) and (0 0 1), respectively. We performed two different simulations with (1) a canonical (number of particles, volume, temperature, namely NVT) ensemble and (2) an isothermal–isobaric (NPT, where P is pressure) ensemble in each direction. Strictly speaking, here the NVT ensemble maintains the compression area only, whereas the length in the compression direction is scaled dynamically. The NPT ensemble means that the compression area can change according to the applied pressure in these two directions (normal to the compression direction). Figure 2 shows variations in principal stresses versus corresponding imposed axial strain (X direction) from 0 to 0.32 for varying temperatures (150 K, 200 K and 250 K) and pressures (40 MPa). Methane hydrate exhibits monotonic strain hardening and has a similar slope to the stress–strain curves regardless of temperature. This phenomenon matches the experimental results (inset of Fig. 2)^11^,^12^. In contrast, the stress of ice shows typical strain softening after it reaches ultimate strength (peak strength) in the first half of the strain domain. The markedly different shapes of the stress–strain curves of normal ice compared with those of the methane hydrate imply that distinct rheological differences exist between the two materials under deformation. These strength behaviours occurred with confinement in a certain finite area. We also allowed the sample to be fully relaxed along the other two directions (namely, the NPT ensemble) (Fig. S2). It is found that the strength of methane hydrate starts to soften at a larger strain than normal ice. This means that the methane hydrate presents strain hardening only if the hydrate is confined in a certain area (or at least when stresses in the normal directions are not fully relaxed). Furthermore, the strength behaviour of the hydrate is the same, regardless of the direction (Fig. S2(a)), whereas that of normal ice is dependent on direction (Fig. S2(b)). This dependence can be addressed by the differences in structures in the three directions of normal ice (Fig. 1, see also Fig. S1). For example, ice is simply a collection of nonplanar “puckered” hexagonal rings in the X-Y and X-Z planes, whereas it is a dense square in the Y-Z plane. Correspondingly, ice is stronger when compressed in the Z direction (Fig. S3).

### Strain Rate Dependency of Strength

**Figure 3:**
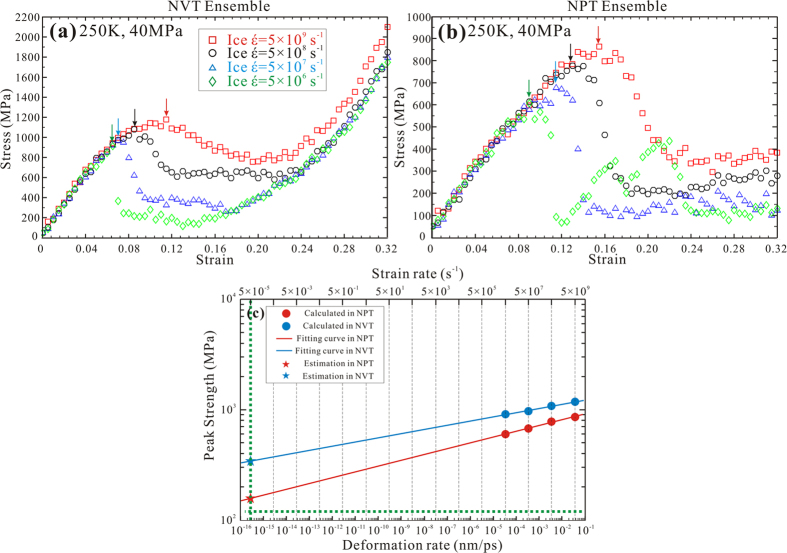
Strain-rate dependence of peak strength for normal ice. Relationships of stress–strain in (**a**) NVT ensemble and (**b**) NPT ensemble. (**c**) Logarithm of evaluated peak strength of normal ice versus logarithm of deformation rate (bottom axis) and strain rate (top axis). The horizontal green dot line represents estimated values (~120 MPa) from experiment at a strain rate of 3.5 × 10^−5 ^s^−1^. All simulation runs were conducted at 250 K and 40 MPa.

We considered the computational capability of the computer and selected four types of applicable strain rates as mentioned in the MD simulation details. The temperature and pressure were set to 250 K and 40 MPa. Figure 3 illustrates the strength behaviour of normal ice depending on loading speed, i.e., strain rate, on the X direction. The slopes of all systems are nearly identical below a certain value, that is, they are within the elastic regime. This indicates that the calculated elastic moduli (presented previously) are unlikely to be affected by the strain rate. On the other hand, the strain rate affects the plastic behaviour of normal ice significantly regardless of the NVT or NPT ensemble. The peak strength and corresponding critical strain point of normal ice are typically reduced with decreasing strain rate. Figure 3(c) shows the estimated peak strength of normal ice with extrapolations to a lower strain rate near the experimental value (3.5 × 10^−5 ^s^−1^). When the strain rate slows to 5 × 10^−5 ^s^−1^, the peak strength of normal ice attained in NPT and NVT ensembles approaches experimental results (~120 MPa). Meanwhile the loading speed influences the mechanical behaviour of methane hydrate remarkably as well in NPT ensemble, but the effect can be negligible in NVT ensemble (Fig. S4). Compared with the strain rate from the simulations, the experimental strain rate is extremely slow, and furthermore, a polycrystalline sample was used in the experiments^11^,^12^. It is remarkable (perhaps incidental) that the extrapolation gave satisfactory results with the same magnitude as the experimental results.

### Mechanisms of Strain Hardening: Function of Guest Methane Molecules

**Figure 4:**
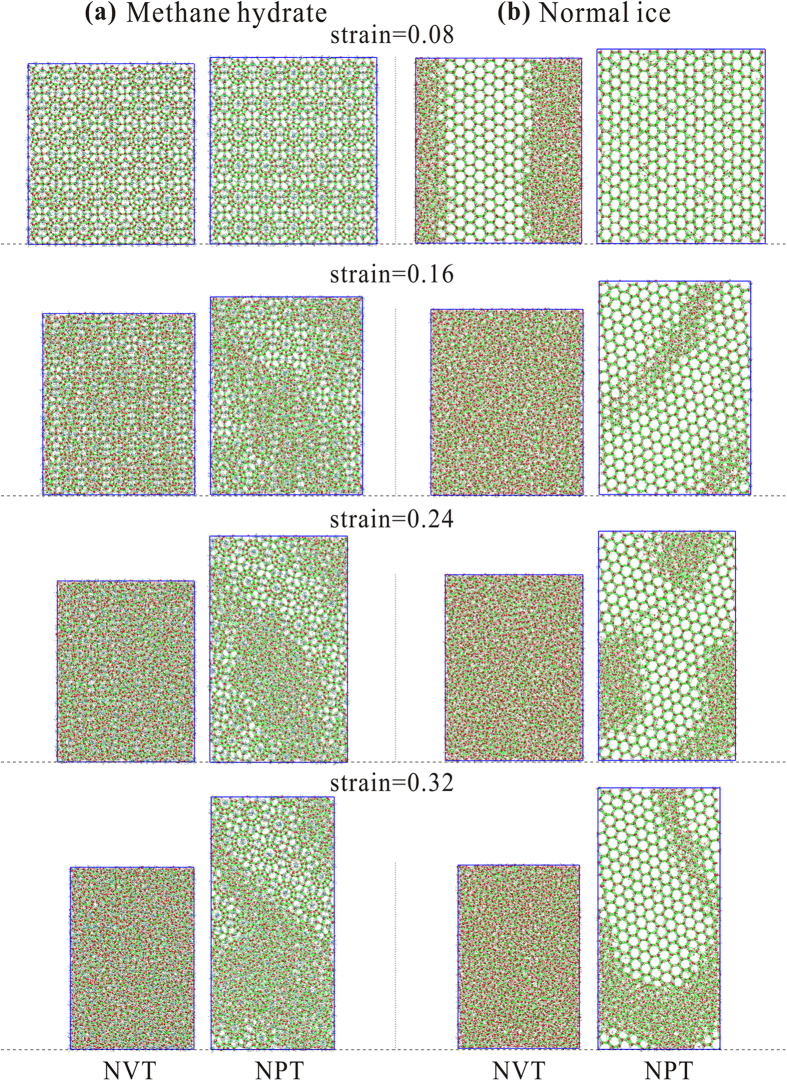
Snapshots of (**a**) methane hydrate and (**b**) normal ice system under compressive deformation test conditions. The first step of presented images is from strain of 0.08. The initial configuration (strain of 0) is shown in Fig. 1.

We present here snapshots of molecular images (Fig. 4) every 0.08 (strain increment) with regard to the deformation process when the strain rate equals 5 × 10^6 ^s^−1^. The crystalline structure cannot be observed clearly in the NVT ensemble except for the first step (strain of 0.08). In contrast, boxes in the NPT ensemble become longer and narrower with time. The hexagonal crystal lattices are retained, even in the last step of normal ice as well as cages in the methane hydrate. This is because the elongation of the box sides in the NPT ensemble leads to a release of rapidly accumulated pressure, whereas in the NVT ensemble, the significantly increasing pressure destroys the crystalline structures. The number of horizontal crystal lattices is also reduced from the beginning to the end in the NPT ensemble. The NVT and NPT situations are considered to be the respective high and low boundaries of mechanical strength because materials cannot completely release the accumulated pressure while bearing the compaction. The experiment should be an expression of a combination of the NVT and NPT effect. The experimental system of the methane hydrate may be more like the NVT ensemble (of our simulations) than that of normal ice. This can be informed by the difference of Poisson’s ratio, where the one of normal ice is slightly higher than that of methane hydrate (Table S3). Furthermore, Peierls stresses of the slip plane^35^ in methane hydrate should be much higher than those in normal ice, since the methane hydrate has much larger crystalline unit cell than normal ice. In fact, the methane hydrate deforms vertically approximately 10^6^ times slower than normal ice^15^. A comparison of steps 3 (strain of 0.24) and 4 (strain of 0.32) of normal ice in the NPT ensemble shows that the hexagonal lattices on the top of the simulation box emerge again after disappearance. This indicates that recrystallization occurred between the two steps. A similar phenomenon was observed for the cage structure of the methane hydrate. This implies that the normal ice and methane hydrate are deformed to reach steady state by grain boundary formation, crystallization, reformation and sliding, as suggested previously^11^,^12^. For the limited size and high strain rate used in this study, we did not observe any dislocation within the samples in our simulations.

**Figure 5:**
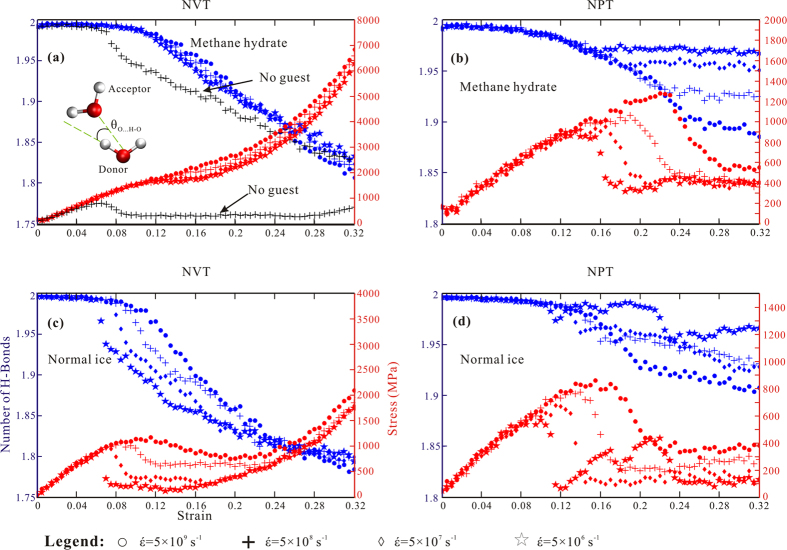
Temporal evolution of H-bond number (in blue) and stress–strain relationship (in red) for a strain from 0 to 0.32. In panel (**a**), black colour-coded marks symbolize a hydrate structure without guest molecules. Circle, 5 × 10^9 ^s^−1^; cross sign, 5 × 10^8 ^s^−1^; diamond, 5 × 10^7 ^s^−1^; and pentagon, 5 × 10^6 ^s^−1^.

The effect of hydrogen bonding^36^,^37^ is discussed to explain the fundamental mechanism. In Fig. 5, the average number of hydrogen bonds (H-bonds) per water molecule in the system is shown together with the stress–strain curves from the beginning (strain of 0) to the end (strain of 0.32) of the compressive process. The H-bonds represent those with a hydrogen-donor–acceptor (HO…O) angle (inset of panel (a)) less than 25° and a distance between the donor and acceptor of less than 0.35 nm. In general, the reduced numbers of H-bonds in NVT are nearly more than two times those in NPT. It can be concluded that (1) hydrogen bonding behaviour is a good indicator of mechanical properties, and (2) all H-bond curves are dependent on strain rate, except for methane hydrate in the NVT ensemble.

First, curves of H-bonds can be divided into three stages according to the slope variation in the NPT ensemble. In the initial stage, the H-bond number maintains an initial level until the strain reaches ~0.08, and then it slopes down to the respective break points, which is defined as the second stage. The slopes’ steepness decreases and they are parallel to each other in the third phase. As the loading speed decreases, break points appear earlier and the reduced numbers of H-bonds decrease. The H-bond curves of the methane hydrate are arranged neatly in comparison to those of normal ice. The break points of the H-bond curves seem to be correlated with the maxima of the stress–strain curves, that is, the break points of H-bonds occur at midpoints between the peak strength and local minima before the levelling off of stress–strain relationships. In the NVT ensemble for normal ice, the H-bond curves also exhibit a three-stage pattern. Disparities are that the curves are apart and parallel to each other in the second stage and converge again in the third stage. Similar to NPT ensembles, the peak strength is more related to the transition from stages one to two.

Second, the methane hydrate in NVT is distinguished from the other three cases because the stress–strain relationships and H-bond behaviours are identical regardless of strain rate. The corresponding H-bond behaviour can be divided into two stages. In the first stage, the H-bond numbers are invariable whereas the stress–strain curves increase with identical slope. Then, H-bond numbers decrease with the same slope whereas the stress increases continuously and monotonically. For comparison, supplementary data (colour-coded in black in Fig. 5(a)) were added to signify the strength behaviour of the hypothetical empty type-I hydrate structure. This structure has identical cavities to the methane hydrate but no guest molecules. Its strength reaches a maximum at a strain of ~0.06, then drops and levels off from a strain of ~0.08. The H-bond number also decreases rapidly compared with the hydrate with guest, although they converge at the end of the deformation process. This phenomenon demonstrates the importance of guest molecules in enhancing hydrate strength.

**Figure 6:**
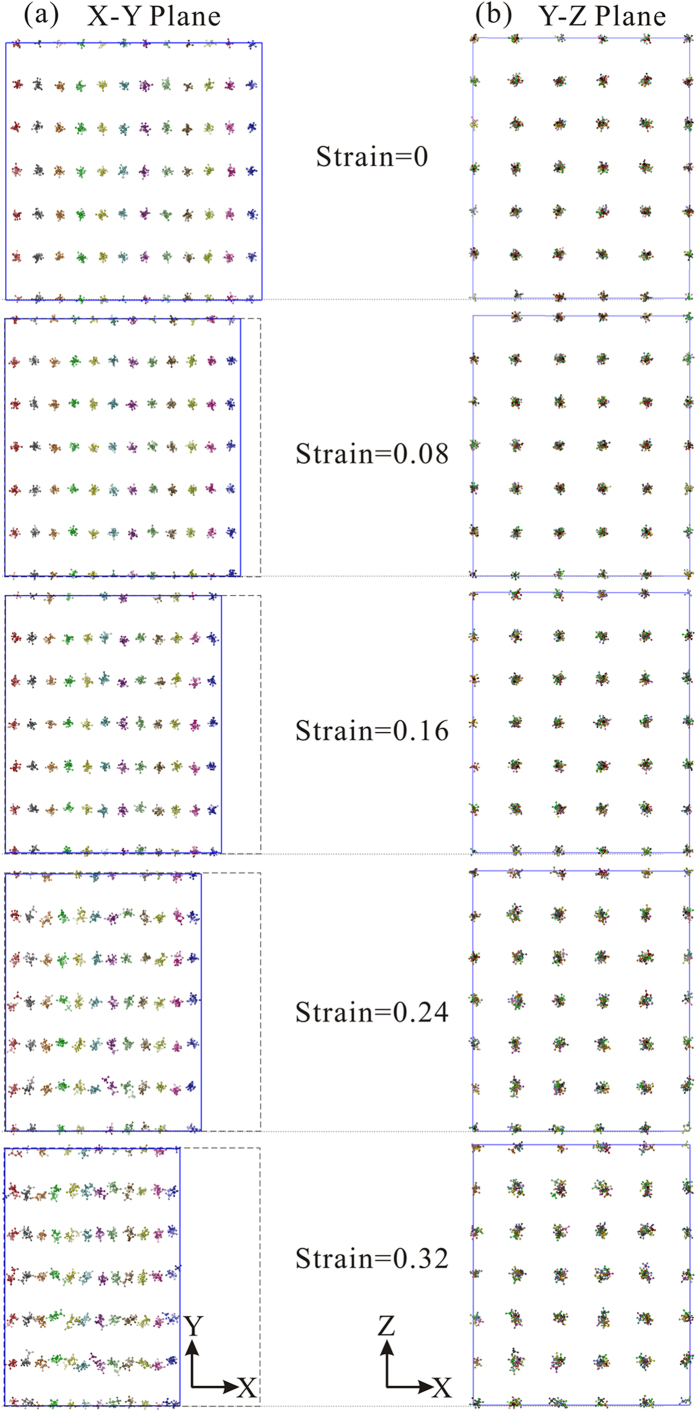
(**a**) Spatial conformation of methane molecules trapped in large cage (5^12^6^2^) on X-Y plane and (**b**) Y-Z plane, which are two of the six from the large cages that are aligned parallel to the X axis.

Figure 6 shows the spatial arrangement of methane molecules from large cages (5^12^6^2^) during deformation with a strain rate of 5 × 10^8 ^s^−1^. These molecules are two of the six from the large cages that are aligned parallel to the X-axis. Every column in the X-Y plane is marked using one colour to observe their interactions during the process. The distance between columns decreases initially in the compressive direction as the strain increases from 0 to 0.16 (Fig. 6(a)). When the space can no longer be compressed, the methane molecules begin to shift up and down with a strain from 0.24 to 0.32. Figure 6(b) shows the conformation on the cross section perpendicular to the compressive direction. The length of the interval between different columns does not change, however, the radii of the methane molecule “cluster” pattern expanded slightly. Therefore, methane molecules do not depart from their original positions and they do not mix with neighbours from adjacent columns.

**Figure 7:**
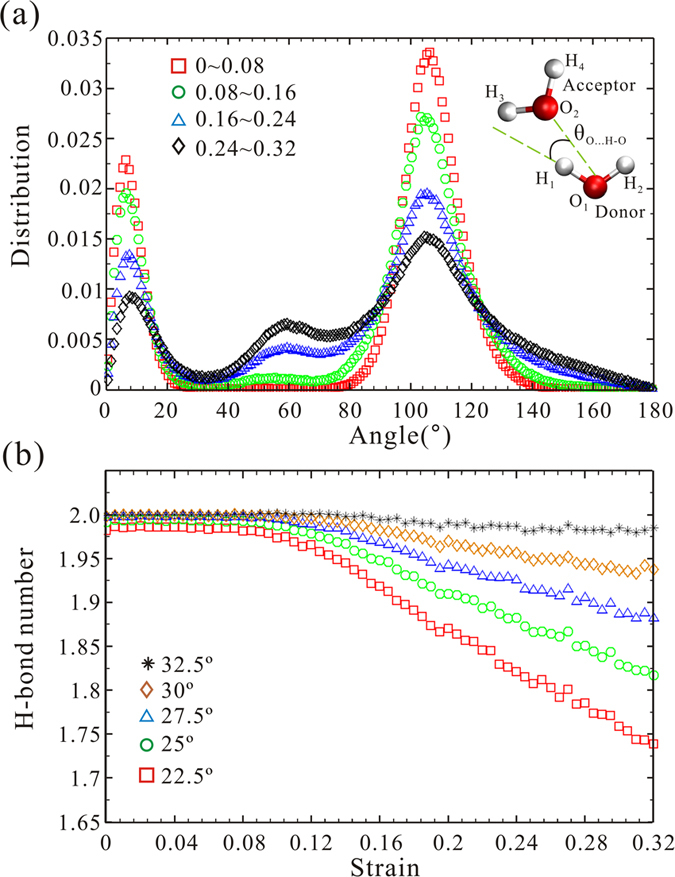
(**a**) Angle distribution of all possible HO…O angles between two hydrogen-bonded water molecules. (**b**) Average number of hydrogen bonds per water molecule with different cutoff of HO…O angles.

Figure 7(a) shows the hydrogen-donor–acceptor (HO…O) distribution angles with a distance between donor and accepter of less than 0.35 nm for hydrate in the NVT ensemble. The strain domain is divided into four intervals (indicated by different colours and marks): ~0–0.08 (red square), ~0.08–0.16 (green circle), ~0.16–0.24 (blue triangle) and ~0.24–0.32 (black diamond). The first group of peaks represents true H-bonds (H_1_O_1_…O_2_) that are related to hydrogen bonding. The second group of peaks is attributed to a significant increase in coordination number, namely interstitial water, which is induced by contraction of the simulation box. The third group of peaks is concerned with other hydrogen atoms of those two hydrogen-bonded water molecules (H_2_O_1_…O_2_, H_3_O_2_…O_1_, H_4_O_2_…O_1_). The red square curve ~(0–0.08) shows a perfect H-bond network and the area under the third peak is three times as large as that of the first peak. The first and third peaks decrease with an increase in strain, whereas the second peak increases. This implies that the number of interstitial water molecules increases with the compressive process. Figure 7(b) illustrates the change in H-bond number depending on defined cutoff HO…O angles, which are related to the first group of peaks shown in Fig. 7(a). Five cases were presented from 22.5° to 32.5° with a 2.5° increase. The reduced number increases with a decrease in cutoff value. When the threshold equals 32.5°, the total number of H-bonds is nearly invariable. This result indicates that the contraction in the simulation box leads to an increase in HO…O angle (namely, the H-bond is more distorted and less directional with increase in strain), which results in an apparent reduction of H-bond number as shown in Fig. 5. Therefore, although the number of interstitial water molecules increases, the number of H-bonds is kept mostly constant because of support from the non-deformable units of the guest molecules.

## Discussion

In summary, methane hydrate and normal ice are “ductile” by grain boundary formation when external axial strain is applied in the NPT ensemble. Methane hydrate shows a strain-hardening phenomenon in the NVT ensemble, whereas normal ice displays peak strength. Based on the analysis, the entrapped methane molecules have no long-distance diffusion when confined in a finite area (NVT ensemble). They serve as non-deformable units to prevent complete failure of the H-bond network in the methane hydrate. The methane hydrate is stronger than normal ice in the NVT and NPT ensembles. However, their overall magnitudes are comparable. This result is the same as that of preceding experimental findings^11^,^12^, but it differs from recent compressive creep tests^14^,^15^, where the methane hydrate is more than 20–40 times stronger than normal ice. In this regard, dislocation motion on the easy glide systems should be investigated^35^,^38^. Finally, methane hydrate is a typical inclusion compound in the H-bond network, and an understanding of the strain-hardening behaviour in this compound may assist further studies of strain hardening in polymers, where the polymer network contributes to strain hardening but requires an explanation of additional mechanisms to understand fully the temperature effects^39^. Our work may also stimulate research into inclusion compounds for desirable strain hardening.

### Theory and Computational Methods

#### Main Theory Applied for Mechanical Properties

Generalized Hooke’s Law^40^ was used to investigate the comparison of the stress–strain relationships between methane hydrate and ice during deformation process. The constitutive equation is as follows:


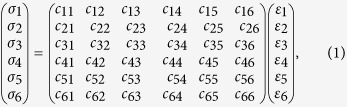


where σ_*i*_ and *ε*_*i*_ represent the stress and strain tensors, respectively. Subscripts (1, 2, 3, 4, 5, 6) denote different directions (XX, YY, ZZ, YZ, ZX, XY). *c*_*ij*_ is the elasticity matrix of materials, which determines the stiffness. This equation accounts for an elastic anisotropy situation with loading in different directions. Elastic constants are obtained from slopes of the stress–strain curves. Once elastic constants are obtained, the bulk modulus K and shear modulus G are calculated from the Voigt and Reuss models^41^, respectively, assuming that strain and stress are uniform throughout the system.

Voigt model:


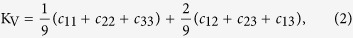



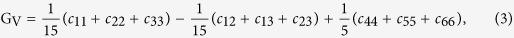


Reuss model:


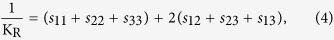



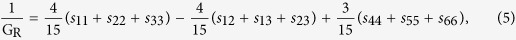


where ***s*** represents the compliance matrix, which is the reverse of the elasticity matrix ***c***. Because the Voigt and Reuss models signify a maximum and minimum value of the moduli, the results are optimized by the Hill average^41^:


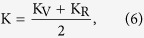



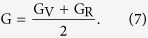


The Hill average results were used as input to calculate Young’s modulus E and Poisson’s ratio *v* as follows^39^:


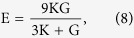



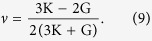


#### MD Simulation Details

We used the GROMACS software package (version 4.5.5)^42^ to perform non-equilibrium MD simulations and model the deformation of methane hydrate and normal ice. The temperature was varied from 150 K to 250 K. The pressure was fixed at 40 MPa. These conditions are close to experimental conditions presented in ref. 11. Nosé–Hoover thermostat^43^ and Parrinello–Rahman pressure coupling^44^ were used for temperature and pressure control, respectively. Particle mesh Ewald summation^45^ was used for the electrostatic interactions. The cutoff value for Van der Waals interactions was set to 1.1 nm. The time step was 1 fs for all simulation runs. The TIP4P/ice water^46^,^47^ and OPLS_AA^48^,^49^ models were used for water and methane, respectively. Initially, all configuration boxes were equilibrated in NPT ensemble for 1 ns. To calculate the elastic moduli, MD simulations were performed in NVT ensemble. A cubic box of methane hydrate that includes 9936 water molecules and 1728 methane molecules was used, and 9216 water molecules were included in the orthorhombic shape of normal ice. The strain was varied every step with a constant strain rate. For calculations of *C*_11_, *C*_12_, and *C*_13_, compressive and tensile axial strain *ε*_1_ (XX direction) was applied on the simulation box. The slopes as defined by σ_1_/*ε*_1_, σ_2_/*ε*_1_, and σ_3_/*ε*_1_ are *C*_11_, *C*_12_, and *C*_13_, respectively. Likewise, when axial strain *ε*_3_ (ZZ direction) was applied, *C*_33_ was obtained from the slope of σ_3_/*ε*_3_. For calculation of *C*_44_, shear strain *ε*_5_ (ZX direction) was applied and the computed data equal σ_5_/*ε*_5_. *ε*_5_ was chosen because we used an orthorhombic box instead of hexagonal one (see Fig. S2 for orientations). The slopes of all systems were obtained within the elastic regime to the strain of ±4% for compressive and tensile test, and ±1% for the shear test (see Fig. S5 for methane hydrate and Fig. S6 for normal ice). The loading speeds were 4 × 10^8 ^s^−1^ and 1 × 10^9 ^s^−1^, respectively. As far as we noted, the slopes are almost identical for all the strain rates that we studied (see Fig. 3(a)). To investigate the strain hardening behaviour and calculate the peak strength of materials, the size of the methane hydrate box was prepared to be almost the same as normal ice. The former contains 8280 water molecules plus 1440 methane molecules, whereas the latter has 9216 water molecules as the calculation for elastic constants. Four different loading speeds were employed: 5 × 10^9 ^s^−1^, 5 × 10^8 ^s^−1^, 5 × 10^7 ^s^−1^ and 5 × 10^6 ^s^−1^. The relationship between peak strength and strain rate (at constant temperature) is then expressed by the following power law relationship of the form^15^:


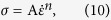


where A is a constant, which is related to temperature and other variables, and *n* is the strain rate sensitivity.

## Additional Information

**How to cite this article**: Jia, J. *et al.* Microscopic Origin of Strain Hardening in Methane Hydrate. *Sci. Rep.* **6**, 23548; doi: 10.1038/srep23548 (2016).

## Supplementary Material

Supplementary Information
