# Supplementary material for: Microscopic Origin of Strain Hardening in Methane Hydrate
Source: Sci Rep. 2016 Mar 24;6:23548. doi: 10.1038/srep23548 (PMC4806379; doi:10.1038/srep23548)
Supplement: Supplementary Information [file srep23548-s1.pdf]

Supplementary Materials

Microscopic Origin of Strain Hardening in Methane Hydrate

Jihui Jia[1], Yunfeng Liang[1,], Takeshi Tsuji[2], Sumihiko Murata[1,*], and Toshifumi Matsuoka[1]*

[1]*Environment and Resource System Engineering, Kyoto University, Kyoto 615-8540, Japan*

[2]*International Institute for Carbon-Neutral Energy Research (I2CNER), Kyushu University, Fukuoka 819-0395, Japan*

[*]Corresponding author: Yunfeng Liang

Postal address:

Kyoto University

Room C1-1-110

Kyotodaigaku-Katsura, Nishikyoku

Kyoto 615-8540, Japan

Tel: +81-75-383-3205

Fax: +81-75-383-3203

Email: y_liang@earth.kumst.kyoto-u.ac.jp

[*]Corresponding author: Sumihiko Murata

Email: murata.sumihiko.6v@kyoto-u.ac.jp

Table S1. Elastic constants of methane hydrate (sI) comparisons with experimental data by Brillouin spectroscopy[18].

Elastic constants (GPa)	Methane hydrate (sI)		
	This study (250K,40MPa)	This study (296K,40MPa)	BS data (296K,40MPa)
C11	15.07	13.89	12.05
C12	9.77	8.72	6.26
C44	3.38	3.23	3.50

Table S2. Elastic constants of normal ice (Ih) and comparisons with experimental data by Brillouin spectroscopy[19].

Elastic constants (GPa)	Normal ice (Ih)		
	This study (250K, 40MPa)	This study (237.65K, 50MPa)	BS data (237.65K, 50MPa)
C11	15.31	15.79	14.73
C12	10.23	10.42	7.63
C13	9.32	9.64	6.19
C33	15.57	15.99	15.89
C44	2.33	2.36	3.13

Table S3. Elastic moduli of methane hydrate (sI) and normal ice (Ih) under 250 K, 40 MPa and comparisons with experimental data by UPT[16].

Moduli (GPa)	Methane Hydrate (SI) at 250 K, 40 MPa		Ice (Ih) at 250 K, 40 MPa	
	This study	UPT data	This study	UPT
K	11.5354	8.27~9.32*	11.5422	8.98~10.12*
G	3.0645	3.50~3.79*	2.5769	3.42~3.71*
v	0.378	0.31~0.33*	0.3961	0.32~0.34*
E	8.4456	8.71~10.49#	7.1952	8.6~10.41#

* represents the data from Helgerud et al., 2009[16] corrected with uncertainty.

# represents the data estimated from bulk and shear modulus of experimental data and corrected with uncertainty.

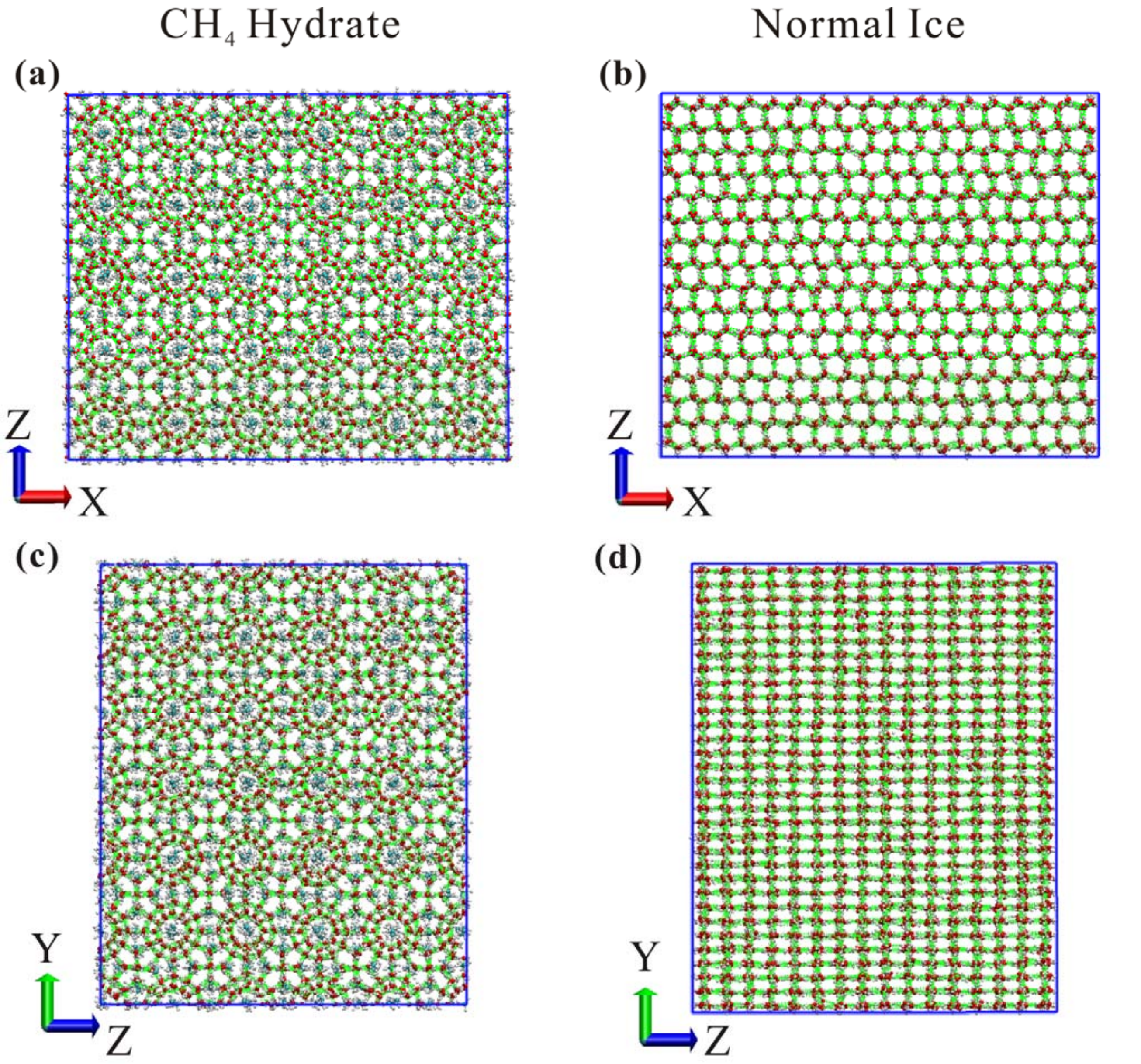

Figure S1. Molecular structures of (a, c) methane hydrate and (b, d) normal ice on different directions. Above are snapshots viewed on X-Z plane of (a) methane hydrate and (b) normal ice. Below are structures of Y-Z plane of (c) methane hydrate and (d) normal ice.

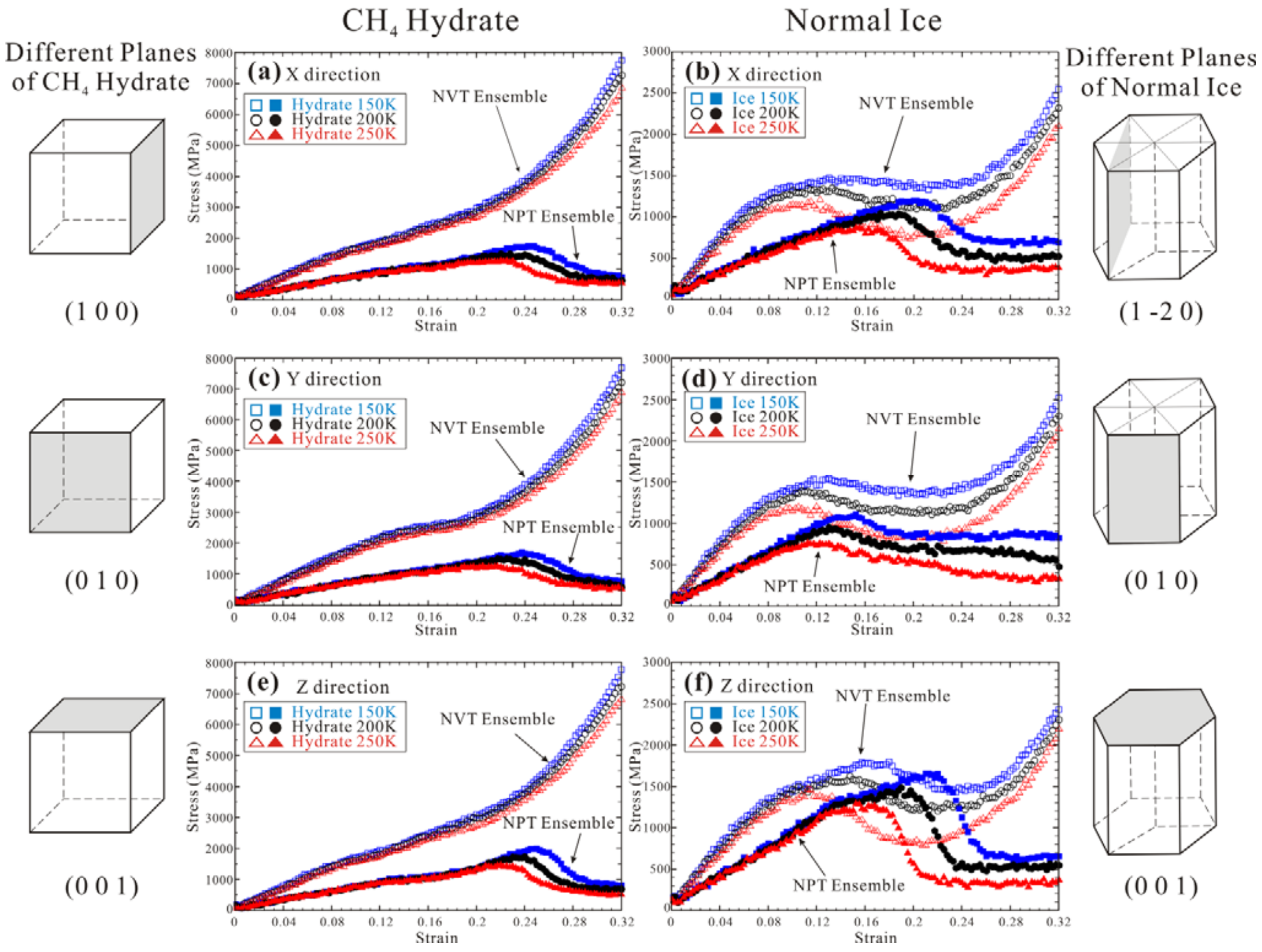

Figure S2. Stress-strain relationships of methane hydrate (a, c, e) and normal ice (b, d, f) along three different directions in NVT and NPT. All the simulation runs were conducted with strain rate of 5×10^9 s^{-1} under 250K and 40 MPa. Data points with open signs are obtained from NVT ensembles. Data points with solid signs are obtained in NPT ensembles. See text for detailed explanation on NVT and NPT ensembles.

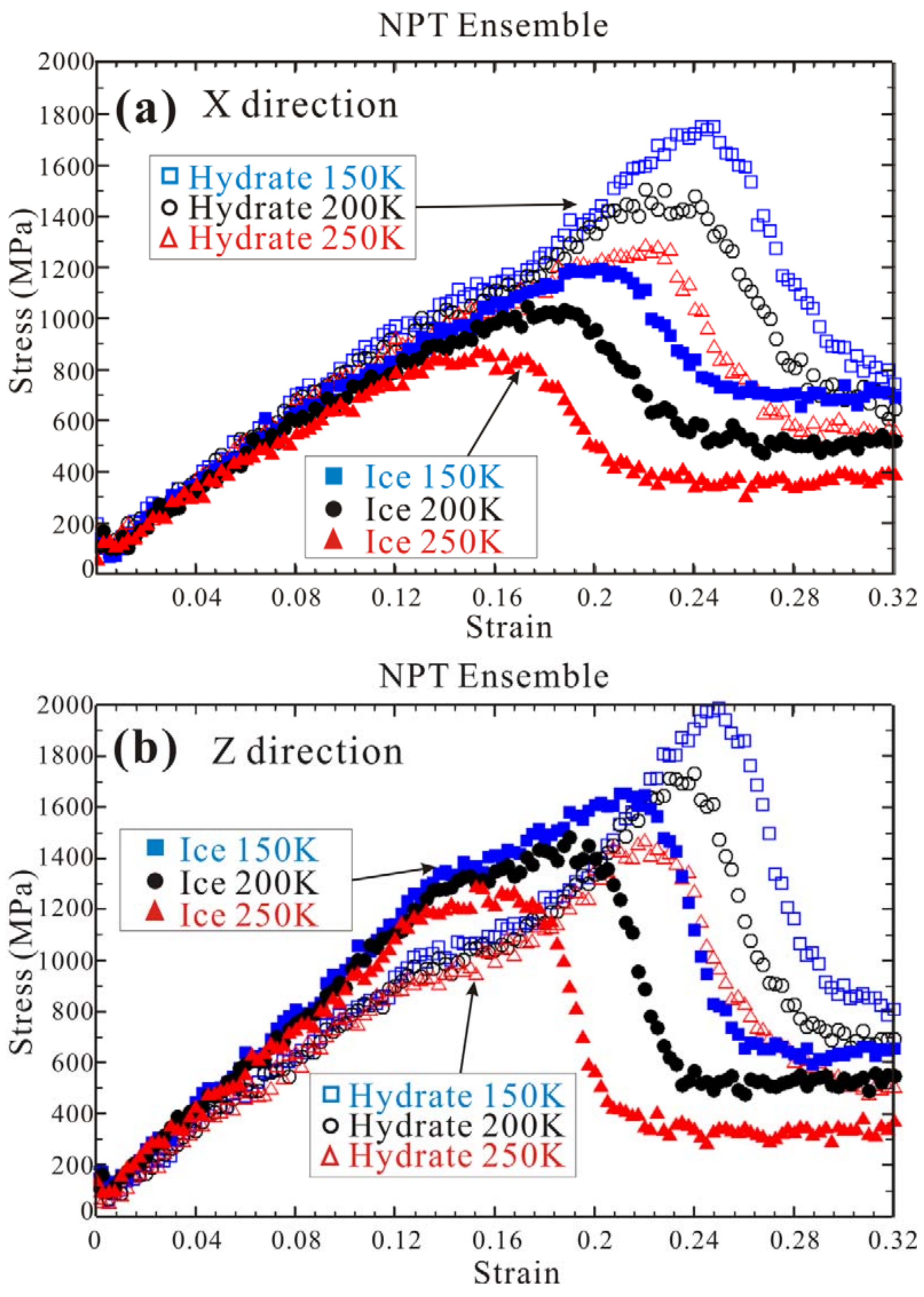

Figure S3. Comparisons of stress-strain relationships between methane hydrate and normal ice along (a) X and (b) Z directions.

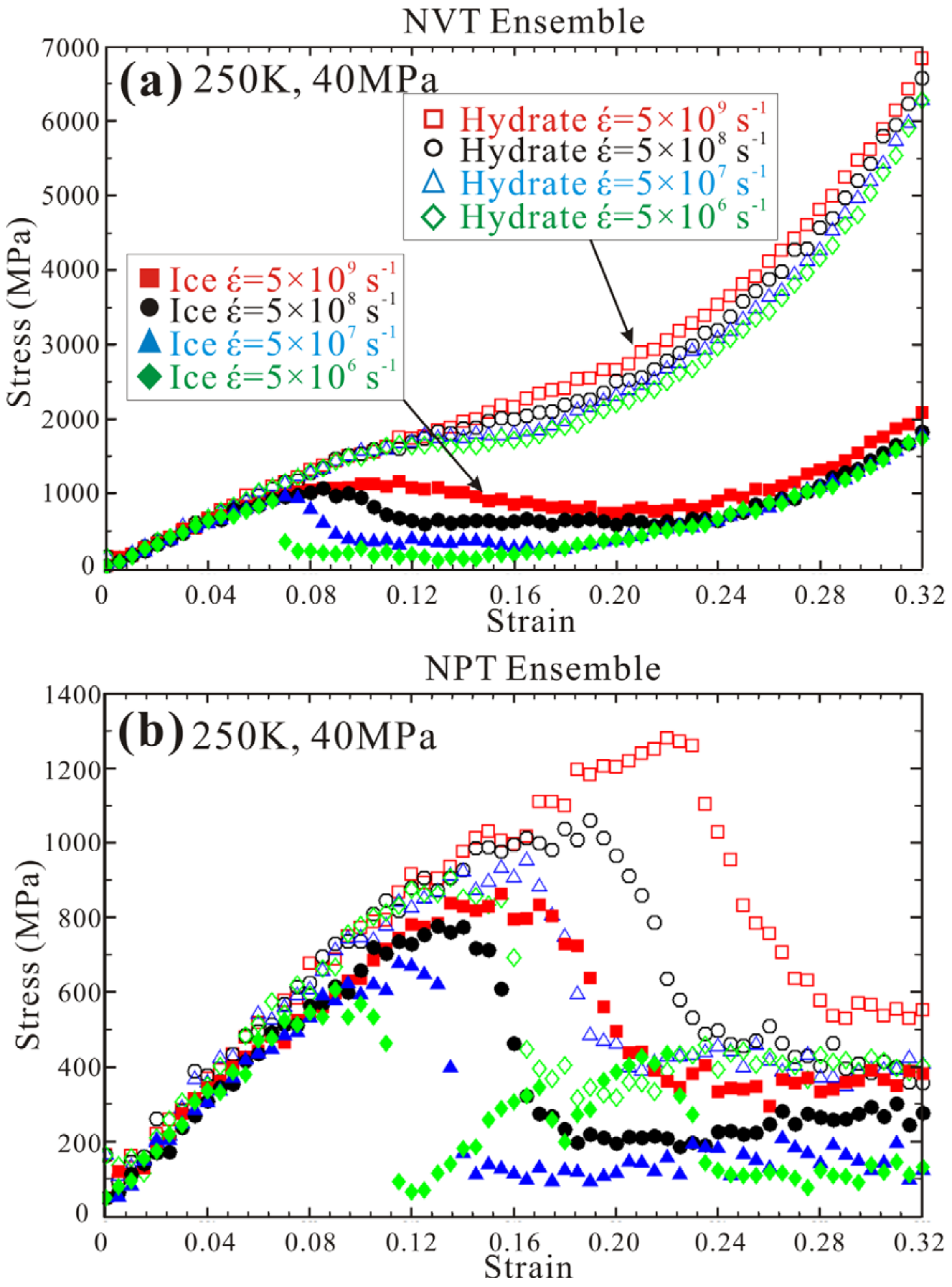

Figure S4. Strain rate dependence of yield strength for methane hydrate and normal ice: (a) relationship of stress-strain in NVT ensemble and (b) in NPT ensemble.

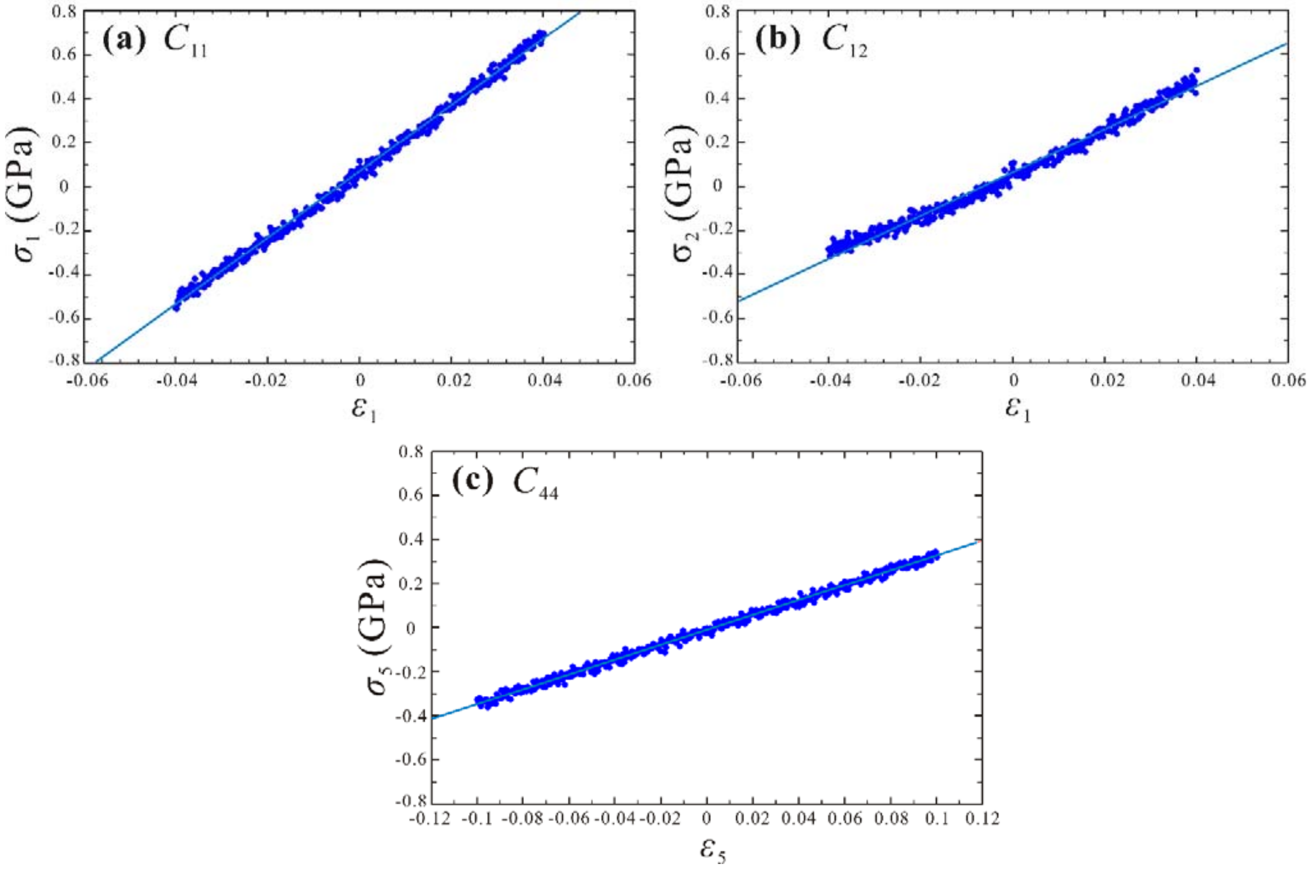

Figure S5. Stress-strain behaviors of gas hydrate with constant strain deformation simulations. C_{11} and C_{12} are from the same axial deformation in XX direction (a) and (b), C_{44} is from the shear deformation (c).

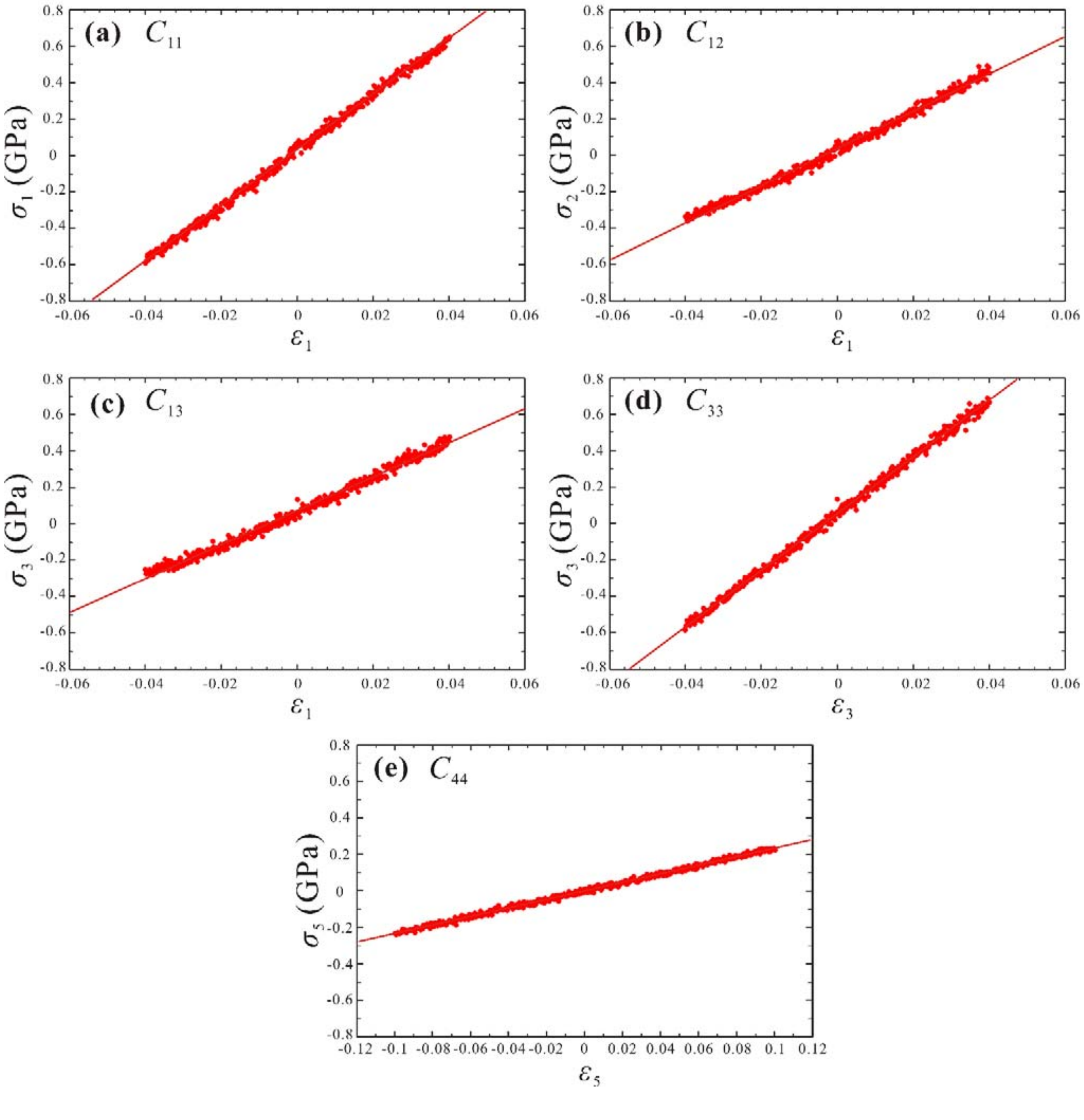

Figure S6. Stress-strain behaviors of gas hydrate with constant strain deformation simulations. C_{11}, C_{12} and C_{13} are from the same axial deformation in XX direction (a), (b) and (c), C_{33} are from the axial deformation in ZZ direction (d), C_{44} is from the shear deformation (e).
